# The vascular flora of the Maritime and Ligurian Alps: Species diversity, functional traits, and conservation status

**DOI:** 10.1002/ecy.70550

**Published:** 2026-10-04

**Authors:** Davide Casalino, Martino Adamo, Gabriele Casazza, Maria Guerrina, Luigi Minuto, Virgile Noble, Marco Mucciarelli

**Affiliations:** ^1^ Department of Earth, Environment and Life Sciences (DISTAV) University of Genova Genoa Italy; ^2^ Department of Life Sciences and Systems Biology (DBIOS) University of Torino Torino Italy; ^3^ Conservatoire Botanique National Méditerranéen (CBNMed) Hyères France

**Keywords:** Alps, biodiversity hotspot, conservation status, functional traits, plant checklist, vascular flora

## Abstract

The three datasets provide occurrences, functional traits, and conservation status for the vascular plants of the Maritime and Ligurian Alps (Italy and France), one of the major biodiversity hotspots in the Mediterranean basin and the primary center of endemism in the Alps. The presence of each taxon was verified through national and international databases, as well as through validation by experts specialized in regional vascular flora and on specific taxonomic groups. The first dataset includes 3521 taxa, comprising 3309 species and 212 subspecies, belonging to 924 genera and 153 families. For each taxon, we provide detailed taxonomical information (phylum, class, family, genus, and taxon rank). To reconcile nomenclatural differences between Italy and France, we standardized the nomenclature according to the International Plant Names Index, reporting national synonyms where available. In the second dataset, we compiled information on the main reproductive, morphological, ecological, and biogeographical features of the taxa. Data were retrieved from Baseflor, FloraVeg.EU, GIFT, SID, and TRY databases and complemented, when necessary, with data from regional and national flora, including Flora d'Italia, Flora Gallica, and Flora Alpina. Information for 21 traits is available for 2032 taxa (57.7%). We successfully assigned at least 19 traits to 842 taxa (23.9%). Another 544 taxa (15.4%) have between 2 and 18 traits, while 99 (2.8%) have between 5 and 10. Fewer than two traits are identified only for three taxa. Two taxa had no trait data available. Life forms and growth forms were the traits with the least missing data (assigned in 99.8% and 99.7% of the taxa, respectively), while seed mass showed the least data (present in 77.9% of the taxa). Plant functional traits are key drivers of ecosystem functioning and vegetation dynamics; therefore, understanding these traits is crucial for predicting how present vascular flora will respond to global warming and anthropogenic pressures in the future. A third dataset was compiled including the IUCN red lists together with international, national, and regional protection with the aim of providing a tool to guide future measures for the conservation of this vascular flora and its habitats, also with the occurrence of invasive or naturalized allochthonous species. Importantly, this will support the development of coordinated management and conservation strategies across two national borders, highlighting the need for a transnational approach to vascular flora and habitat conservation in the Maritime and Ligurian Alps. The three datasets are designed to be continuously updated, through their integration with new data on species occurrence, functional traits, and conservation status as these become available to the scientific community. Together, they provide a robust baseline for future research on plant evolution and vegetation dynamics and responses to global changes. Furthermore, these datasets will enhance data consistency and support informed decision‐making and policy development in response to the emerging threats to plants and habitats posed by global warming. By making these data openly available, we adhere to FAIR principles (Findable, Accessible, Interoperable, and Reusable), supporting open research in conservation and macroecology. The data are available for reuse under CC BY 4.0 licensing. Please cite the data release and this data paper when reusing the dataset.

## CONFLICT OF INTEREST STATEMENT

The authors declare no conflicts of interest.

## Supporting information


Data S1.

## Data Availability

Data are available as in Data S1. These data are also available at Figshare at https://doi.org/10.6084/m9.figshare.29369915.

