# Supplementary material for: The vascular flora of the Maritime and Ligurian Alps: Species diversity, functional traits, and conservation status
Source: Ecology. 2026 Oct 4;107(10):e70550. doi: 10.1002/ecy.70550 (PMC13635772; doi:10.1002/ecy.70550)
Supplement: Supplementary file 1 [file ECY-107-e70550-s001.zip › Metadata_S1.pdf]

Data Paper published in *Ecology*, an Ecological Society of America journal

Metadata S1

The vascular flora of the Maritime and Ligurian Alps: Species diversity, functional traits, and conservation status

Davide Casalino, Martino Adamo, Gabriele Casazza, Maria Guerrina, Luigi Minuto, Virgile Noble, Marco Mucciarelli

Class I. Data Set Descriptors

A. Data set identity:

Species diversity, functional traits, and conservation status of the vascular flora of the Maritime and Ligurian Alps

B. Data set identification code: https://doi.org/10.6084/m9.figshare.29369915

C. Data set description:

A harmonized checklist of vascular plant species and subspecies occurring in the Ligurian Alps, Maritime Alps, and Alps and Prealps of Provence, coupled with functional trait data and protection status information.

1. Originators:

Author's names:

Davide Casalino[1], Martino Adamo[2], Gabriele Casazza[1], Maria Guerrina[1], Luigi Minuto[1], Virgile Noble[3], Marco Mucciarelli[2]

Institutional addresses:

1- University of Genova, Department of Earth, Environment and Life Sciences (DISTAV), Corso Europa 26, 16132 Genova, Italy
2 - University of Torino, Department of Life Sciences and Systems Biology (DBIOS), Viale Pier Andrea Mattioli 25, 10125 Torino, Italy
3- Conservatoire Botanique National Méditerranéen (CBNMed), 34 Av. Gambetta, 83400 Hyères, France

2. Abstract:

The three data sets provide occurrences, functional traits and conservation status for the vascular plants of the Maritime and Ligurian Alps (Italy and France), one of the major biodiversity hotspots in the Mediterranean basin and the primary centre of endemism in the Alps. The presence of each taxon was verified through national and international databases, as well as through validation by experts specialized in regional vascular flora and on specific taxonomic groups. The first data set includes 3,521 taxa, comprising 3,309 species and 212 subspecies, belonging to 924 genera and 153 families. For each taxon, we provide detailed taxonomical information (phylum, class, family, genus, and taxon rank). To reconcile nomenclatural differences between Italy and France, we standardized the nomenclature according to the International Plant Names Index, reporting national synonyms where available. In the second data set, we compiled information on the main

reproductive, morphological, ecological and biogeographical features of the taxa. Data were retrieved from Baseflor, FloraVeg.EU, GIFT, SID, and TRY databases and complemented, when necessary, with data from regional and national flora, including Flora d'Italia, Flora Gallica and Flora Alpina. Information for 21 traits is available for 2,032 taxa (57.7%). We successfully assigned at least 19 traits to 842 taxa (23.9%). Another 544 taxa (15.4%) have between two and 18 traits, while 99 (2.8%) have between five and 10. Fewer than two traits are identified only for three taxa. Two taxa had no trait data available. Life forms and growth forms were the traits with the least missing data (assigned in 99.8% and 99.7% of the taxa, respectively), while seed mass showed the least data (present in 77.9% of the taxa). Plant functional traits are key drivers of ecosystem functioning and vegetation dynamics; therefore, understanding these traits is crucial for predicting how present vascular flora will respond to global warming and anthropogenic pressures in the future. A third data set was compiled including the IUCN red lists together with international, national, and regional protection with the aim of providing a tool to guide future measures for the conservation of this vascular flora and its habitats, also with the occurrence of invasive or naturalized allochthonous species. Importantly, this will support the development of coordinated management and conservation strategies across two national borders, highlighting the need for a transnational approach to vascular flora and habitat conservation in the Maritime and Ligurian Alps. The three data sets are designed to be continuously updated, through their integration with new data on species occurrence, functional traits, and conservation status as these become available to the scientific community. Together, they provide a robust baseline for future research on plant evolution and vegetation dynamics and responses to global changes. Furthermore, these data sets will enhance data consistency and support informed decision-making and policy development in response to the emerging threats to plants and habitats posed by global warming. By making these data openly available, we adhere to FAIR principles (Findable, Accessible, Interoperable, and Reusable), supporting open research in conservation and macroecology. The data are available for reuse under CC BY 4.0 licensing. Please cite the data release and this data paper when reusing the dataset.

D. Key words:

Alps; biodiversity hotspot; conservation status; functional traits; plant checklist; vascular flora

Class II. Research origin descriptors

A. Overall project description:

1. Identity:

National Biodiversity Future Center (NBFC) - Spoke3 Research unit of Torino

2. Originators:

Prof Marco Mucciarelli
Dr. Martino Adamo

Address:
Consiglio Nazionale delle Ricerche, Piazza Marina, 61, 90133 Palermo, Italia.
University of Torino, Department of Life Sciences and Systems Biology (DBIOS), Viale Pier Andrea Mattioli 25, 10125 Torino, Italy

3. Period of study: June 2023 - February 2026

4. Objectives:
NBFC aims to monitor, preserve and restore marine, terrestrial and urban ecosystems in Italy. This data set is part of the monitoring action, with the aim to set a baseline for future activities of monitoring, research and conservation of the Natural Heritage.

5. Abstract:
The National Biodiversity Future Center (NBFC) is the first Italian research centre dedicated to biodiversity valorization and conservation. NBFC was born in 2022 as a project based on EU Commission funds. It is structured around an innovative model that includes a central HUB and eight "spokes", each focusing on specific areas of research and development, with the aim of addressing global challenges related to biodiversity and sustainability.
Within the framework of the National Biodiversity Future Center (NBFC), arising from the applied botany research groups of the Universities of Torino and Genova, the idea emerged to develop the first publicly available checklist of the vascular flora of the Ligurian and Maritime Alps. The idea of a checklist was then expanded to this data set representing a contribution to the knowledge of one of the most species-rich floras in Europe. The initiative was developed within NBFC-Spoke 3 (Land Biodiversity) of the University of Torino, in close collaboration with the University of Genova and the Conservatoire Botanique National Méditerranéen.

6. Sources of funding:
National Recovery and Resilience Plan (NRRP), Mission 4 Component 2 Investment 1.4 Call for tender No. 3138 of 16 December 2021, rectified by Decree n.3175 of 18 December

2021 of Italian Ministry of University and Research funded by the European Union – NextGenerationEU; Award Number: Project code CN_00000033, Concession Decree No. 1034 of 17 June 2022 adopted by the Italian Ministry of University and Research, CUP D13C22001350001, Project title "National Biodiversity Future Center - NBFC"

7. Site description

The southernmost part of the Alps is one of the main biodiversity hotspots in the Mediterranean Basin (Medail & Verlasque, 1997), where Mediterranean and Alpine climates meet (Ozenda & Borel, 2000). This area, located on the border between north-western Italy and south-eastern France (Medail & Verlasque, 1997), is also one of the most important biogeographical regions in Europe (Casazza et al., 2005) thanks to the high concentration of endemisms, which makes it the main centre of endemisms in the Alps (Pawlowski, 1970).
The region is geologically complex (Bertotti et al., 2006) due to its tectonic (Rosenbaum & Lister, 2005) and lithological characteristics, including calcareous and siliceous outcrops, as well as ophiolitic substrates. Its geomorphology ranges in elevation from sea level up to 3,297 m a.s.l. at Mt. Argentera.
To define the boundaries of our work, we started from the Ligurian and Maritime Alps hotspot, already investigated by botanists, researchers, professors and technicians in the past, and we extended the study area to the surrounding Alps characterised by Mediterranean climate too.
For this reason we used the SOIUSA classification of Alps, selecting sections 1. Ligurian Alps; 2. Maritime Alps; 3. Alps and Prealps of Provence (Marazzi, 2005) (**Figure 1**) for a total surface of 16,631 km2 (calculated with the open source GIS QGIS).
The southern part of the study area coincides with the coastal line between Savona and Nice, where the limit goes to the Vallon des Trigands where the Préalpes de Grasse starts. These, with the limits of the Préalpes de Digne, Préalpes de Vaucluse and the Alpes de Provence, are the western part of our study area. Following the river L'Ubaye from Barcelonnette to Bois de la Silve, the northern limit goes down to Argentera, Pianche and then goes east until Borgo San Dalmazzo, where Maritime Alps finishes and Ligurian Alps starts. The eastern boundary follows the boundaries of the Ligurian Prealps and Marguareis Alps, to the Cadibona Pass.

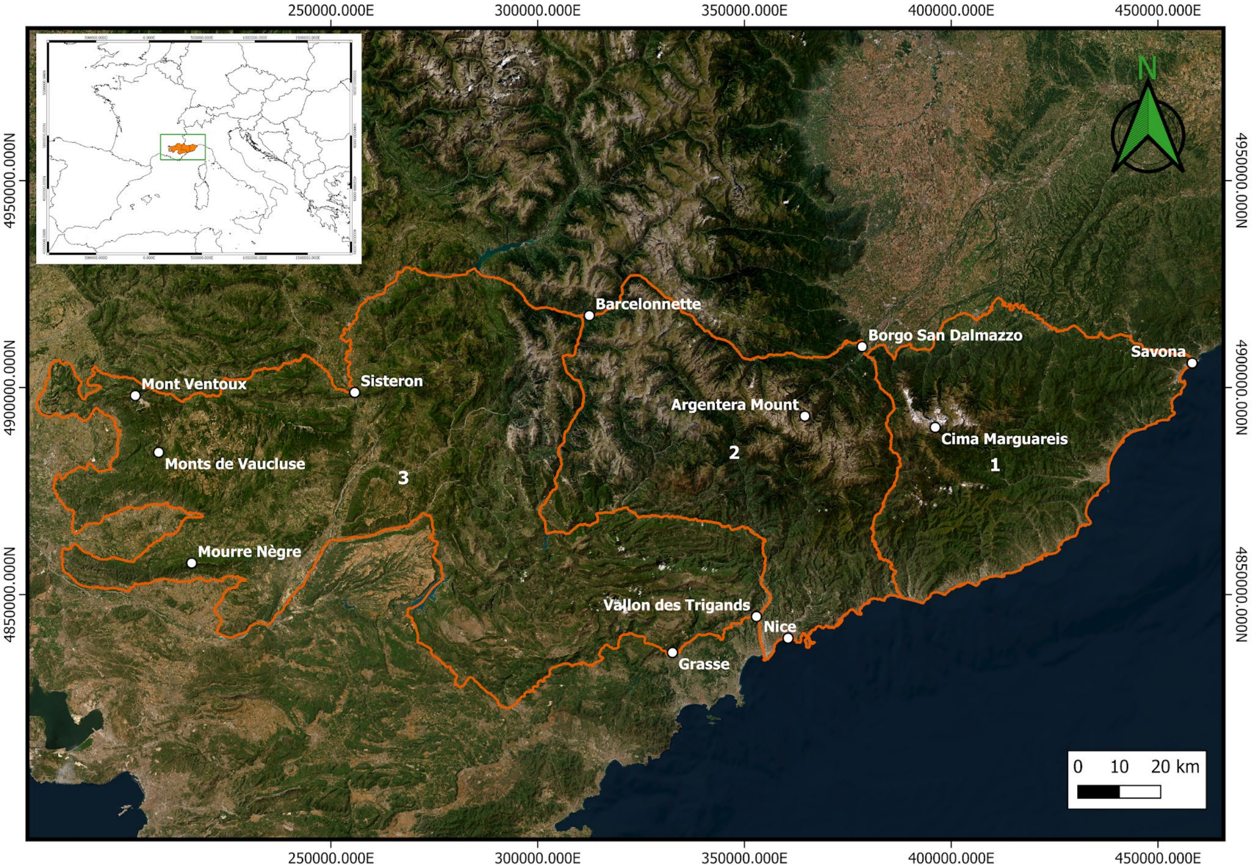

Figure 1 Geographical context of the study area. Position across the Italian and French boundary (top-left box), and detail of the southern part of the Alps (top-right box). In orange the boundaries of the Alpine sectors included in the study: 1. Ligurian Alps; 2. Maritime Alps; 3. Alps and Prealps of Provence. Map created using QGIS and the ESRI Satellite tiles for the background representation.

8. Experimental design and research methods

a. Species occurrence:

The checklist was compiled thanks to several years of experience in floristic studies focused on the study region (e.g. Casazza et al., 2005; Adamo et al., 2021; Guerrina et al., 2022; Adamo et al., 2023; Mucciarelli et al., 2025; Casazza et al., 2026). Taxa occurrence was assessed through a four-step validation process designed to ensure taxonomic consistency and reliable presence data. First, the presence or absence of each taxon was evaluated at the national level using Plants of the World Online (POWO, 2026). Second, occurrences were verified at regional and local scales using Tela Botanica (www.tela-botanica.org) for French taxa, and FlorItaly - Portal to the Flora of Italy (https://dryades.units.it/floritaly/) based on the checklist of the vascular flora native to Italy (Bartolucci et al., 2024) and the checklist of the vascular flora alien to Italy (Galasso et al., 2024) and their updates for Italian taxa. Third, georeferenced occurrence records were

examined using the curated Silene Nature database. Finally, all records were manually reviewed and validated by the authors or expert collaborators, who have specific knowledge in the vascular flora of the Ligurian and Maritime Alps or in targeted taxonomic groups (e.g. Orchidaceae, *Fritillari*a, *Narcissus*, *Tephroseris*).

b. Taxonomic harmonization:

For each taxon, we provide detailed taxonomical information (phylum, class, family, genus, and taxon rank). Taxa accepted names were obtained and updated according to the International Plant Name Index by using the R package WorldFlora v 1.14.5 (Kindt, 2020; last access 14/01/2026). When appropriate, we included synonyms for the French and Italian taxa using Tela Botanica or Silene Nature, and the updated checklist of the vascular flora native to Italy as references for the two countries.

c. Functional traits database:

Once the checklist was outlined, we collected publicly available plant traits from several sources following a hierarchical prioritization:

I. TRY – Plant Trait Database (Kattge et al., 2011)
II. Baseflor (Julve, 1998)
III. GIFT – The Global Inventory of Floras and Traits (Weigelt et al., 2020)
IV. FloraVeg.EU (Chytrý et al., 2024)
V. TelaBotanica (https://www.tela-botanica.org/)
VI. National and regional flora (Aeshimann, 2004; Tison & De Foucault, 2014; Pignatti et al., 2017)

Seed weights have been sourced from SID - Seed Information Database [14]. To integrate the different data sets, all data were preliminary harmonized by using the IPNI nomenclature. Data wrangling, extraction and table merging was performed in the R environment by using functions from the tidyverse package v2.0.0.
TRY data set references are accessible at doi:https://doi.org/10.6084/m9.figshare.29369915 in Supplementary materials 2.pdf

d. Conservation status database:

Data regarding the conservation status of the species, including the protection level, were manually compiled in accordance with the Habitat Directive, alongside national (Italian and French) or regional legislations, including Regione Piemonte, Regione Liguria and Provence-Alpes-Côte d'Azur (PACA). The conservation status at the global level was automatically obtained with the R package rredlist v0.7.1 from the IUCN Red List of Threatened Species. The conservation status for France and Italy was manually filled using the national Red List manuals (Orsenigo et al., 2021; IUCN, 2025).
Taxon endemicity represents a key characteristic of the vascular flora of the study area, which constitutes the main centre of endemism within the European Alps. Most endemic

taxa occurring in this region are shared between Italy and France, while a smaller number are restricted to a single country. Because administrative boundaries do not reflect biogeographical patterns, endemicity was assessed by deliberately disregarding national borders and referring exclusively to the entire study area. A taxon was therefore considered endemic if its natural distribution is limited to the Ligurian and Maritime Alps. Conversely, it was considered subendemic if its natural distribution is mainly confined to, but not exclusively within, the Ligurian and Maritime Alps.
This column of the database was compiled by hand from bibliography (Aeshimann, 2004; Tison & De Foucault, 2014; Pignatti et al., 2017; Bertolli et al., 2024).
Moreover, a "Status" column had been added to report if the species is native, naturalized or invasive (Galasso et al., 2024)

9. Project personnel:

Experts involved into the species validation:

- Dr. Jacopo Calevo for Orchidaceae
- Prof. Giuseppina Barberis for the species of Liguria

Class III. Data set status and accessibility

A. Status

Complete; subject to future updates

1. Latest update: 18th June 2026
2. Latest archive date: 18th June 2026
3. Metadata status: 18th June 2026
4. Data verification: 12nd May 2026

B. Accessibility

1. Storage location and medium:
   Data are available with this publication in *Ecology* and in the Figshare repository at https://doi.org/10.6084/m9.figshare.29369915

2. Contact persons:
   Martino Adamo
   Address: University of Torino, Department of Life Sciences and Systems Biology (DBIOS), Orto Botanico di Torino, Viale Pier Andrea Mattioli 25, 10125 Torino, Italy

Email: martino.adamo@unito.it
Phone: +39 011 6705557

Gabriele Casazza
Address: University of Genova, Department of Earth, Environment and Life Sciences (DISTAV), Corso Europa 26, 16132 Genova, Italy
Email: gabriele.casazza@unige.it

3. Copyright restrictions: The data are available for reuse under CC BY 4.0 licensing. Please cite the data release and this data paper when reusing the dataset.

4. Proprietary restrictions: none

a. Release date: NA
b. Citations:

Casalino D., Adamo M., Casazza G., Guerrina M., Minuto L., Noble V., Mucciarelli M. (2026). The vascular flora of the Maritime and Ligurian Alps: Species diversity, functional traits, and conservation status. *Ecology*.

Casalino, Davide; Adamo, Martino; Casazza, Gabriele; Guerrina, Maria; Minuto, Luigi; Noble, Virgile; et al. (2025). Checklist of the Vascular Flora of Maritime and Ligurian Alps: the dataset on Species Diversity, Functional Traits, and Conservation Status. Figshare. https://doi.org/10.6084/m9.figshare.29369915.v2

c. Disclaimer(s):
The present data set represents the current state of knowledge for the study area and is intended as a dynamic resource. It may be updated and refined in the future following well-documented and properly communicated suggestions submitted to the designated contact persons.

Class IV. Data structural descriptors

A. Data set file

1. Identity (Unique file names or codes):

MACvp_funtionaltraits.txt
MACvp_conservation.txt
MACvp_systematic.txt
functions.R
Rscript.R

The data set consists of three ‘txt’ format files: MACvp_systematic.txt, MACvp_functionaltraits .txt and MACvp_conservation. Are tab-delimited text files and can be uploaded to most statistical software or database and data set management systems. Special characters in scientificNameAuthorship such as accents on the authors names (e.g., ü in Günther, š in Bureš or é in Thévenau) had been changed to simple characters to avoid problems with character encoding. These files can be imported with LibreOffice and OpenOffice selecting ‘tab’ for field delimitation.
For managing the data sets, we provide an RStudio project named “MACvp_project.Rproj” that can be open with RStudio. Inside the project we provide two folders: “script” and “data”.
In “data” folder there are the three data set “MACvp_systematic.csv”, “MACvp_functionaltraits.csv” and “MACvp_conservation.csv”; in the “script” folders there are two more files: ‘functions.R’ contains the functions that can be used, and ‘Rscript.R’, to load and merge the three data sets, and for searching taxa. These codes had been tested in RStudio-2024.12.1-563 version under Windows version 10 and 11.

2. Size:

Table 1 Files description and size in terms of number of records and variables included.

**File name**	**Description**	**Records**	**Variables**
MACvp_funtionaltraits.txt	data set of the species' functional traits	3521	22
MACvp_conservation.txt	data set of the species' conservation status	3521	14
MACvp_systematic.txt	Checklist of the species including updated taxonomy	3521	15
Rfunctionr.R	R function to explore the data sets	NA	NA
Rscript.R	A detailed R script to enhance the user experience	NA	NA

3. Format and storage mode:
Uncompressed tab-delimited table in standard ASCII format, saved as *.txt files.

4. Header information:

a. <u>MACvp_conservation.txt</u>
   i. checklistID
   ii. IUCN_Global
   iii. IUCN_FR

iv. IUCN_IT
v. DirHabitat
vi. DirHabitat_Annex
vii. FrLaw_95
viii. LiguriaLaw_84
ix. LiguriaLaw_Annex
x. PiedmontLaw_82
xi. PACALaw_94
xii. PACALaw_Article
xiii. Endemism
xiv. Status

b. MACvp_funtionaltraits.txt
i. checklistID
ii. inflorescence
iii. floral_Sex
iv. pollination
v. fruit_Type
vi. dispersal_Mode
vii. flower_Color
viii. flowering_Start
ix. flowering_End
x. sizeRange_cm
xi. life_Form
xii. growth_Form
xiii. seedMass_gr
xiv. Chorology
xv. vegetation_Belt
xvi. macro_Habitat
xvii. EIV_Light
xviii. EIV_Temp
xix. EIV_Moisture
xx. EIV_Reaction
xxi. EIV_Nutrients
xxii. EIV_Salinity

c. MACvp_systematic.txt
i. checklistID
ii. taxonID
iii. taxonomicStatus

iv. scientificName
v. scientificNameAuthorship
vi. syn_FlorItaly
vii. syn_TelaBot
viii. phylum
ix. class
x. order
xi. family
xii. genus
xiii. species
xiv. subspecies
xv. taxonRank

B. Variables description

Variable name	data set	Description	Unit	Data type	Range
checklistID	all	unique identifier of the MACvp entries. This is common across the three data set to ensure correspondence.	NA	numeric	1 to 3521
taxonID	checklist	Taxon ID as reported in World Flora Online		character	
taxonomicStatus	checklist	Taxonomic status as reported in World Flora Online [Accepted, Unchecked, Not in WOF/TPL]	NA	character	NA
scientificName	checklist	taxon name without author	NA	character	NA
syn_FlorItaly	checklist	most recent synonym from Portal to the Flora of Italy	NA	character	NA
syn_TelaBot	checklist	last per the most recent synonym from TelaBotanica	NA	character	NA
phylum	checklist	phylum of the taxon	NA	character	NA
class	checklist	class of the taxon	NA	character	NA

order	checklist	order of the taxon	NA	character	NA
family	checklist	family of the taxon	NA	character	NA
genus	checklist	genus of the taxon	NA	character	NA
taxonRank	checklist	specification if the taxon is species or subspecies SPECIES, SUBSPECIES	NA	character	NA
scientificNameAuthorship	checklist	name of the author/authors of the taxon	NA	character	NA
IUCN_Global	conservation	Conservation status according to IUCN categories for Global Red Lists [EX, EW, RE, CR, EN, VU, NT, LC, DD, NE]	NA	categorical	NA
IUCN_FR	conservation	Conservation status according to IUCN categories for French Red Lists [EX, EW, RE, CR, EN, VU, NT, LC, DD, NE]	NA	categorical	NA
IUCN_IT	conservation	Conservation status according to IUCN categories for Italian Red Lists [EX, EW, RE, CR, EN, VU, NT, LC, DD, NE]	NA	categorical	NA
DirHabitat	conservation	Presence or absence in Habitat Directive 43/92 [TRUE/FALSE]	NA	boolean	NA
DirHabitat_Annex	conservation	Annex of the Habitat Directive where the taxon is present [II, IV, V]	NA	categorical	NA
FrLaw_95	conservation	Presence or absence in French Law 31/08/1995 [TRUE/FALSE]	NA	boolean	NA
LiguriaLaw_84	conservation	Presence or absence in Ligurian law 9/1984 [TRUE/FALSE]	NA	boolean	NA
LiguriaLaw_Annex	conservation	Annex where the taxon is present [A, B]	NA	categorical	NA
PiedmontLaw_82	conservation	Presence or absence in Piedmont law 32/1982 (art. 15) [TRUE/FALSE]	NA	boolean	NA
PACALaw_94	conservation	Presence or absence in Provence-Alpes-Côte-d’Azur law 9/5/1994 [TRUE/FALSE]	NA	boolean	NA

PACALaw_Articl	conservation	Annex in law 9/5/1994 where the taxon is present [1, 2, 3, 4, 5, 6]	NA	categorical	NA
Endemism	conservation	Exclusive presence in the study area? or with a limited range close to our study area [0 = Not endemic, 1 = Sub endemic, 2 = Endemic]	NA	categorical	NA
Status	conservation	If the taxon is native, naturalized or invasive in the study area. [Native, Naturalized, Invasive]		categorical	
Inflorescence	traits	how flowers aggregate , flower and inflorescence morphology [Capitate inflorescences, Capitula, Capitula of capitula, Capituliform racemes, Compound cymes, Compound racemes, Compound umbels, Corymb of capitula, Corymb racemes, Corymbs, Cyathium, Cymes of capitula, Cymes of glomerules, Cymes of spikes, Dichasium, Double scorpioid cyme, Glomerules, Helicoid cyme, Inflorescences, Panicle, Panicle of corymbs, Panicle of spikes, Panicula, Pauci-flowered, Pleiochasium, Raceme of cymes, Raceme of helicoid cymes, Raceme of scorpioid cymes, Racemes, Racemes of capitula, Racemes of spikes, Racemes of umbels, Scorpioid cyme, Simple umbel, Solitary flowers, Spadix, Spiked inflorescences, Spikes, Spikes of capitula, Spikes of cymes, Umbel, Umbellate cymes, Umbels, Umbels of capitula, Umbels of spikes, Verticels of umbels, Other]	NA	categorical	NA
floral_Sex	traits	sexual strategies [Dioecious; Monoecious; Polygamonoecious; Polygamodioecious; Trimonoecy; other]	NA	categorical	NA
pollination	traits	the mode by which the pollen is transferred to pollinate the flowers [Anemophily; Autogamy; Entomophily; Hydrophily; Other]	NA	categorical	NA
fruit_Type	traits	fruit morphology and classification [Achene; Berry; Capsule; Caryopsis; Cypsela; Drupe; Follicle; Legume; Mericarp; Nut; Pyxidium; Samara; Silicula; Siliqua; Other]	NA	categorical	NA
dispersal_Mode	traits	fruit or seed dispersal strategies [Anemochory; Autochory; Hemerochory; Hydrochory; Ombrochory; Zoochory]	NA	categorical	NA
flower_Color	traits	background color (sepals or tepals) [Black; Blue; Brown; Green; Orange; Pink; Purple; Red; White; Yellow]	NA	categorical	NA
flowering_Start	traits	beginning of flowering period (month within a year 1-12) [Jan; Feb; Mar; Apr; May; Jun; Jul; Aug; Sep; Oct; Nov; Dec]	NA	categorical	NA
flowering_End	traits	ending of flowering period (month within a year 1-12) [Jan; Feb; Mar; Apr; May; Jun; Jul; Aug; Sep; Oct; Nov; Dec]	NA	categorical	NA
sizeRange_cm	traits	height's average of the plant	cm	numeric	0 - 10000

life_Form	traits	Raunkiaer's biological form of the taxon [Chamaephyte; Geophyte; Hemicryptophyte; Hydrophyte; Phanerophyte; Therophyte]	NA	categorical	NA
growth_Form	traits	growth forms of the taxon [Graminoid; Herb; Shrub; Subshrub; Tree]	NA	categorical	NA
seedMass_gr	traits	1000 seeds weight	g	numeric	0.0004 - 12976.2200
Chorology	traits	plant distribution	NA	categorical	NA
vegetation_Belt	traits	altitude range	m a.s.l.	numeric	0 - 3297
macro_Habitat	traits	typical conditions where the taxon grows [1 = (aquatic environment s.l. (permanent, temporary); 2 = (± nitrophilous terrestrial, natural or anthropogenic vegetation); 3 = (rocky outcrops, sand, travertine deposits, cliffs, screes, quarries); 4 = (spring, streams); 5 = (swamps, bogs); 6 = (meadows, pastures, sparse grasslands, snow valleys, herbaceous forest margins); 7 = (moors, mega moors, garrigues, shrublands. s.l.); 8 = (thickets s.l.); 9 = (woods)]	NA	categorical	NA
EIV_Light	traits	Ellenberg Indicator Value on light radiation [1,2,3,4,5,6,7,8,9,10,11,12]	NA	categorical	NA
EIV_Temp	traits	Ellenberg Indicator Value on heat [1,2,3,4,5,6,7,8,9,10,11,12]	NA	categorical	NA
EIV_Moisture	traits	Ellenberg Indicator Value on soil moisture or water availability [1,2,3,4,5,6,7,8,9,10,11,12]	NA	categorical	NA
EIV_Reaction	traits	Ellenberg Indicator Value on soil reaction [1,2,3,4,5,6,7,8,9]	NA	categorical	NA
EIV_Nutrients	traits	Ellenberg Indicator Value on nutrients [1,2,3,4,5,6,7,8,9]	NA	categorical	NA
EIV_Salinity	traits	Ellenberg Indicator Value on salinity [1,2,3]	NA	categorical	NA

C. Data anomalies and limitations:

Despite the data set providing a comprehensive overview of the species occurring in the study area, some limitations still persist and need further clarifications. In particular, 523

taxa need to be reviewed to clarify whether they have been misreported in the area or whether they have become extinct. Moreover, 66 taxa were considered problematic from a taxonomic point of view (e.g., different Author names for the same taxon) and were excluded from the checklist (data not shown). Given that the nomenclatural status of apomictic species is subject to frequent change, the Authors are aware that the binomial reported in the checklist may be rapidly revised, as taxonomic updates are ongoing and therefore prone to frequent variation. Many species are characteristic or unique to this area which has likely hindered the collection of functional traits: more than 14% of the data are missing (reported as NA), especially for the Ellenberg indices and seed mass (**Figure 2**), due to the limited research on these taxa (Adamo et al., 2021). Conservation status has been assessed at the national or regional level rather than being specific to the ALMsl area and, therefore, an effort will be undertaken to address and mitigate this bias.

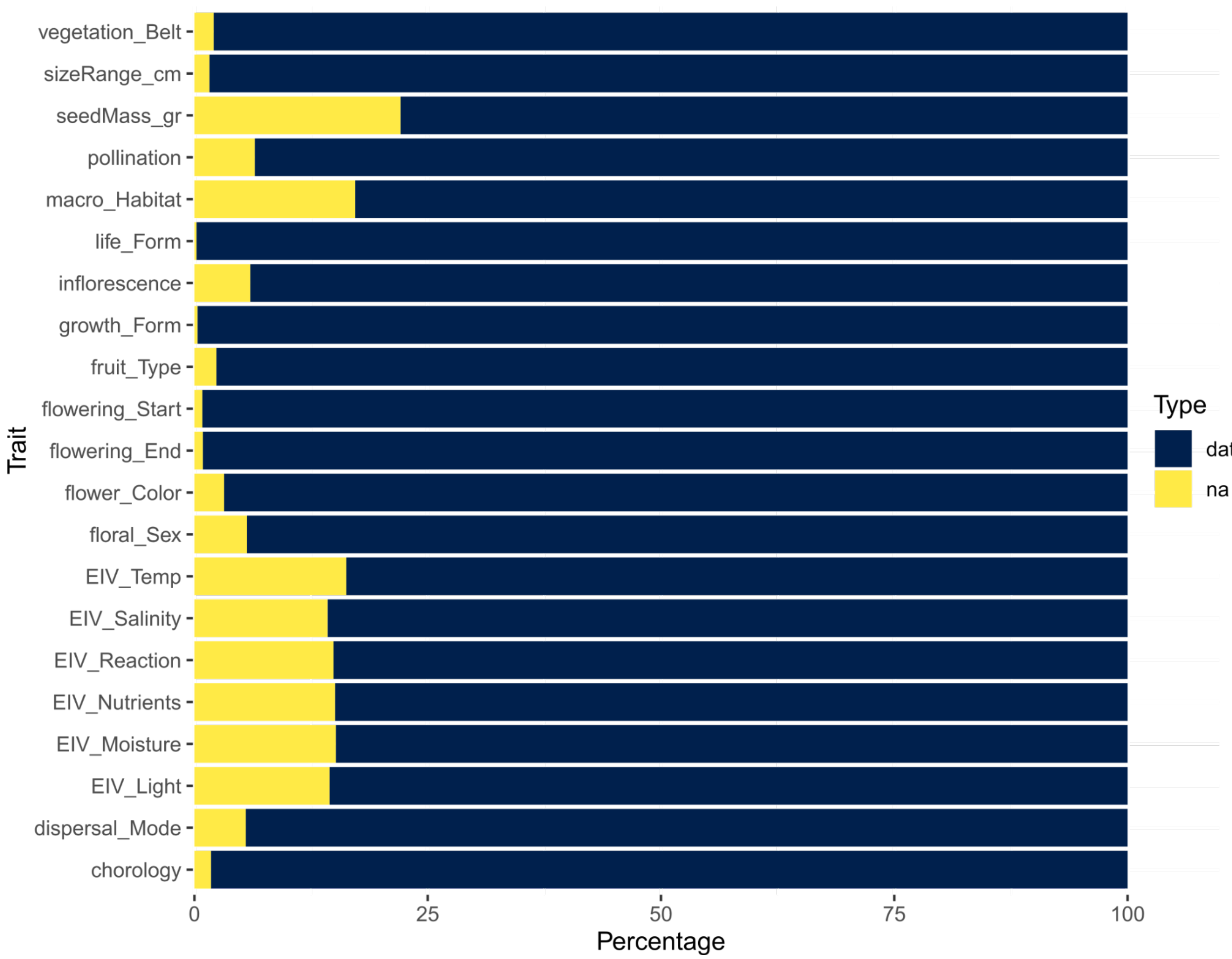

Figure 2 - Proportion of missing values (NAs) across functional traits. Purple sections of the stacked horizontal barplots represent the number of taxa missing this trait. Percentages displayed to the right of each plot indicating the proportion of NAs for each trait.

Class V. Supplemental descriptors

A. Data acquisition

1. Data forms or acquisition methods:

Data were acquired through the compilation and integration of existing sources, including published literature, curated online databases, and expert knowledge. Species occurrence records, functional traits, and conservation information were extracted from authoritative sources, taxonomically harmonized, and recorded in digital tabular formats. No standardized field data forms or automated data loggers were used specifically for this data set.

2. Location of completed data forms:

The data set was compiled from existing bibliographic sources, online databases, and expert validation. No original field data forms were produced specifically for this data set.

3. Data entry verification procedures, and quality control procedures:

Data entries were mainly generated through automated extraction and processing using R scripts, ensuring reproducibility and minimizing transcription errors. Manual data entry was limited to the initial raw species list, the assignment of species endemicity, and the compilation of information on inclusion in international, national, and regional protection laws.

Taxonomic consistency was verified using automated name harmonization tools (see Methods section). All critical or ambiguous taxonomic names were subsequently checked manually against authoritative online resources, including World Flora Online (WFO), Plants of the World Online (POWO) and the International Plant Names Index (IPNI). Additional consistency checks were performed to verify variable formats, missing values (NAs), and logical coherence across the different data tables prior to final export.

B. Related materials:

R script and functions for data merging and querying.

C. Computer programs and data-processing algorithms:

All data processing was performed in R environment version 4.4.2 (Rcore Team, 2024) by using tidyverse package for data wrangling (Wickham et al., 2019), WorldFlora to taxonomy harmonization, taxize to associate Linnean ranks to species, rredlist to automatically query the IUCN database for multiple species.

D. Archiving

1. Archival procedures:
data sets were archived as non-proprietary, plain text files (tab-delimited format) to minimize the risk of file corruption and to ensure long-term accessibility and full transferability across operating systems and software platforms. All files are accompanied by detailed metadata describing structure, variables, and formats, and are deposited in a public data repository to support long-term preservation and reuse.

2. Redundant archival sites:
In addition to the primary public repository (Figshare, see Open Research statement) and this data paper release in *Ecology*, local backup copies of the data set are maintained by the authors on institutional storage systems. The data paper release in *Ecology* and the Figshare repository serve as the authoritative archival sources for long-term access.

E. Publications and results:
Conference contributes:

1. Casalino D., Adamo M., Casazza G., Guerrina M., Minuto L., Mucciarelli M. 2024. “Database della flora delle Alpi Liguri, Marittime, Alpi e Prealpi di Provenza”. Società Italiana di Biogeografia, XLIII Congresso. Grosseto, Italy. October 25 – 27.

2. Casalino D., Adamo M., Casazza G., Guerrina M., Minuto L., Mucciarelli M., 2024. “Checklist della flora delle Alpi Liguri, Marittime, Alpi e Prealpi di Provenza: un database per la raccolta di tratti funzionali”. Società Botanica Italiana Sezione Regionale Ligure, Riunione Scientifica. Genoa, Italy. November 15.

3. Casalino D., Adamo M., Casazza G., Guerrina M., Minuto L., Mucciarelli M. 2025. "Mediterranean Alps" checklist of vascular plants: the first data collection on alpine mediterranean flora functional trait. Alpine Flora: Biodiversity and Conservation International Conferences. Cogne, Italy. June 5-7.

Literature Citations

Adamo, M., M. Chialva, J. Calevo, F. Bertoni, K. Dixon, and S. Mammola. 2021. Plant scientists' research attention is skewed towards colourful, conspicuous and broadly distributed flowers. Nature Plants 7:574–578.

Adamo, M., K. Skokanová, J. Bobo-Pinilla, E. Giaccone, J. Peñas de Giles, M. Mucciarelli. 2023. Molecular evidence and environmental niche evolution at the origin of the disjunct distribution in three mountain endemic Tephroseris (Asteraceae) of the Mediterranean basin. Alpine Botany 133:117-133.

Aeschimann, D., K. Lauber, and D. André Michel. 2004. Flora alpina: atlante delle 4500 piante vascolari delle Alpi. Zanichelli.

Bartolucci, F., L. Peruzzi, G. Galasso, A. Alessandrini, N. M. G. Ardenghi, G. Bacchetta, E. Banfi, G. Barberis, L. Bernardo, D. Bouvet, M. Bovio, G. Calvia, M. Castello, L. Cecchi, E. Del Guacchio, G. Domina, S. Fascetti, L. Gallo, G. Gottschlich, R. Guarino, L. Gubellini, N. Hofmann, M. Iberite, P. Jiménez-Mejías, D. Longo, D. Marchetti, F. Martini, R. R. Masin, P. Medagli, S. Peccenini, F. Prosser, F. Roma-Marzio, L. Rosati, A. Santangelo, A. Scoppola, A. Selvaggi, F. Selvi, A. Soldano, A. Stinca, R. P. Wagensommer, T. Wilhalm, and F. Conti. 2024. A second update to the checklist of the vascular flora native to Italy. Plant Biosystems - An International Journal Dealing with all Aspects of Plant Biology 158:219–296.

Bartolucci F, Domina G, Buccomino G, Ciaschetti G, Conti F, Costanza N, De Luca A, Del Guacchio E, Falcinelli F, Forte L, Galasso G, Ganz C, Iamonico D, Lonati M, Marengo G, Mei G, Nota G, Orsenigo S, Pazienza G, Pellegrino G, Pinzani L, Stinca A, Tavilla G, Tilia A, Tomaselli V, Tondi G, Venanzoni R, Lastrucci L, 2024 - Notulae to the Italian native vascular flora: 18. Italian Botanist 18: 97-108. https://doi.org/10.3897/italianbotanist.18.140958

Bertolli, A., M. Adorni, A. Alessandrini, S. Andreatta, N. Ardenghi, C. Argenti, E. Bona, M. Bovio, R. Dellavedova, B. Gallino, and others. 2024. Flora endemica nel Nord Italia. Athesia; Fondazione Museo Civico Rovereto.

Bertotti, G., P. Mosca, J. Juez, R. Polino, and T. Dunai. 2006. Oligocene to Present kilometres scale subsidence and exhumation of the Ligurian Alps and the Tertiary Piedmont Basin (NW Italy) revealed by apatite (U–Th)/He thermochronology: correlation with regional tectonics. Terra Nova 18:18–25.

Casazza, G., G. Barberis, and L. Minuto. 2005. Ecological characteristics and rarity of endemic plants of the Italian Maritime Alps. Biological Conservation 123:361–371.

Casazza, G., M. Adamo, M. Guerrina, L. Minuto, and M. Mucciarelli. 2026. Incorporating Refugia Capacity in Assessing Plant Climate Vulnerability: A Study Case on Alpine Endemics. Ecology and Evolution 16: e72555.

Chytrý, M., M. Řezníčková, P. Novotný, D. Holubová, Z. Preislerová, F. Attorre, I. Biurrun, P. Blažek, G. Bonari, D. Borovyk, N. Čeplová, J. Danihelka, D. Davydov, P. Dřevojan, N. Fahs, R. Guarino, B. Güler, S. M. Hennekens, R. Hrivnák, V. Kalníková, V. Kalusová, T. Kebert, I. Knollová, K. Knotková, D. Koljanin, A. Kuzemko, J. Loidi, Z. Lososová, C. Marcenò, G. Midolo, D. Milanović, L. Mucina, P. Novák, E. V. Raab-Straube, K. Reczyńska, J. H. J. Schaminée, P. Štěpánková, K. Świerkosz, J. Těšitel, T. Těšitelová, L. Tichý, D. Vynokurov, W. Willner, and I. Axmanová. 2024. FLORAVEG . EU — An online database of European vegetation, habitats and flora. Applied Vegetation Science 27:e12798.

Galasso G, Conti F, Peruzzi L, Alessandrini A, Ardenghi NMG, Bacchetta G, Banfi E, Barberis G, Bernardo L, Bouvet D, Bovio M, Castello M, Cecchi L, Del Guacchio E, Domina G, Fascetti S, Gallo L, Guarino R, Gubellini L, Guiggi A, Hofmann N, Iberite M, Jiménez-Mejías P, Longo D, Marchetti D, Martini F, Masin RR, Medagli P, Musarella CM, Peccenini S, Podda L, Prosser F, Roma-Marzio F, Rosati L, Santangelo A, Scoppola A, Selvaggi A, Selvi F, Soldano A, Stinca A, Wagensommer RP, Wilhalm T, Bartolucci F (2024) A second update to the checklist of the vascular flora alien to Italy. Plant Biosystems, https://doi.org/10.1080/11263504.2024.2320129

Galasso G, Domina G, Bacchetta G, Barberis D, Bartolucci F, Cancellieri L, Ceschin S, Ciaramella D, Croce A, Cuena-Lombraña A, Del Guacchio E, Di Lernia D, Fois M, Fontana D, Franzoni J, Giacò A, Laface VLA, Lallai A, Lonati M, Lupoletti J, Maccioni A, Mascia F, Mei G, Morabito A, Musarella CM, Pelella E, Pica A, Pinzani L, Podda L, Stinca A, Varricchione M, Lastrucci L, 2024b - Notulae to the Italian alien vascular flora: 17. Italian Botanist 17: 43-53. https://doi.org/10.3897/italianbotanist.17.126768

Guerrina, M., S. Theodoridis, L. Minuto, E. Conti, and G. Casazza. 2022. First evidence of post-glacial contraction of Alpine endemics: Insights from *Berardia subacaulis* in the European Alps. Journal of Biogeography 49:79–93.

IUCN 2026, https://www.iucnredlist.org/.

Julve, P. Baseflor. Index Botanique, Ecologique et Chorologique de la Flore de France http://philippe.julve.pagesperso-orange.fr/baseflor.xlsx (1998).

Kattge, J., S. Díaz, S. Lavorel, I. C. Prentice, P. Leadley, G. Bönisch, E. Garnier, M. Westoby, P. B. Reich, I. J. Wright, J. H. C. Cornelissen, C. Violle, S. P. Harrison, P. M. Van Bodegom, M. Reichstein, B. J. Enquist, N. A. Soudzilovskaia, D. D. Ackerly, M. Anand, O. Atkin, M. Bahn, T. R. Baker, D. Baldocchi, R. Bekker, C. C. Blanco, B. Blonder, W. J. Bond, R. Bradstock, D. E. Bunker, F. Casanoves, J. Cavender-Bares, J. Q. Chambers, F. S. Chapin Iii, J. Chave, D. Coomes, W. K. Cornwell, J. M. Craine, B. H. Dobrin, L. Duarte, W. Durka, J. Elser, G. Esser, M. Estiarte, W. F. Fagan, J. Fang, F. Fernández-Méndez, A. Fidelis, B. Finegan, O. Flores, H. Ford, D. Frank, G. T. Freschet, N. M. Fyllas, R. V. Gallagher, W. A. Green, A. G. Gutierrez, T. Hickler, S. I. Higgins, J. G. Hodgson, A. Jalili, S. Jansen, C. A. Joly, A. J. Kerkhoff, D. Kirkup, K. Kitajima, M. Kleyer, S. Klotz, J. M. H. Knops, K. Kramer, I. Kühn, H. Kurokawa, D. Laughlin, T. D. Lee, M. Leishman, F. Lens, T. Lenz, S. L. Lewis, J. Lloyd, J. Llusià, F. Louault, S. Ma, M. D. Mahecha, P. Manning, T. Massad, B. E. Medlyn, J. Messier, A. T. Moles, S. C. Müller, K. Nadrowski, S. Naeem, Ü. Niinemets, S. Nöllert, A. Nüske, R. Ogaya, J. Oleksyn, V. G. Onipchenko, Y. Onoda, J. Ordoñez, G. Overbeck, W. A. Ozinga, S. Patiño, S. Paula, J. G. Pausas, J. Peñuelas, O. L. Phillips, V. Pillar, H. Poorter, L. Poorter, P. Poschlod, A. Prinzing, R. Proulx, A. Rammig, S. Reinsch, B. Reu, L. Sack, B. Salgado-Negret, J. Sardans, S. Shiodera, B. Shipley, A. Siefert, E. Sosinski, J. -F. Soussana, E. Swaine, N. Swenson, K. Thompson, P. Thornton, M. Waldram, E. Weiher, M. White, S. White, S. J. Wright, B. Yguel, S. Zaehle, A. E. Zanne, and C. Wirth. 2011. TRY – a global database of plant traits. Global Change Biology 17:2905–2935.

Marazzi, S. 2005. Atlante orografico delle Alpi. SOIUSA. Suddivisione orografica internazionale unificata del Sistema Alpino. Priuli & Verlucca, Scarmagno.

Médail, F., and R. Verlaque. 1997. Ecological characteristics and rarity of endemic plants from southeast France and Corsica: Implications for biodiversity conservation. Biological Conservation 80:269–281.

Mucciarelli, M., A. Infuso, M. Rix, J. Nilson, J. Calevo, and M. F. Fay. 2025. 1154. Fritillaria of the Maritime Alps: Liliaceae. Curtis's Botanical Magazine 42:355–368.

Orsenigo, S., G. Fenu, D. Gargano, C. Montagnani, T. Abeli, A. Alessandrini, G. Bacchetta, F. Bartolucci, A. Carta, M. Castello, and others. 2021. Red list of threatened vascular plants in Italy. Plant Biosystems 155:310–335.

Ozenda, P., and J.-L. Borel. 2000. An ecological map of Europe: why and how? Comptes Rendus de l'Académie des Sciences - Series III - Sciences de la Vie 323:983–994.

Pawlowski, B. 1970. Remarques sur l'endemisme dans la flore des Alpes et des Carpates. Vegetatio 21:181–243.

Pignatti, S., R. Guarino, M. La Rosa, and others. 2017. Flora d'italia. Edagricole Calderini Milano.

POWO, 2026. https://powo.science.kew.org/.

R Core Team. 2024. R: A Language and Environment for Statistical Computing. R Foundation for Statistical Computing, Vienna, Austria.

Rosenbaum, G., and G. S. Lister. 2005. The Western Alps from the Jurassic to Oligocene: spatio-temporal constraints and evolutionary reconstructions. Earth-Science Reviews 69:281–306.

Tison, J.-M., and B. de Foucault. 2014. Flora gallica: flore de France. Biotope.

Weigelt, P., C. König, and H. Kreft. 2020. GIFT – A Global Inventory of Floras and Traits for macroecology and biogeography. Journal of Biogeography 47:16–43.

Wickham, H., M. Averick, J. Bryan, W. Chang, L. McGowan, R. François, G. Grolemund, A. Hayes, L. Henry, J. Hester, M. Kuhn, T. Pedersen, E. Miller, S. Bache, K. Müller, J. Ooms, D. Robinson, D. Seidel, V. Spinu, K. Takahashi, D. Vaughan, C. Wilke, K. Woo, and H. Yutani. 2019. Welcome to the Tidyverse. Journal of Open Source Software 4:1686.
